# Mechanisms, contexts and points of contention: operationalizing realist-informed research for complex health interventions

**DOI:** 10.1186/s12874-018-0641-4

**Published:** 2018-12-27

**Authors:** James Shaw, Carolyn Steele Gray, G. Ross Baker, Jean-Louis Denis, Mylaine Breton, Jennifer Gutberg, Gaya Embuldeniya, Peter Carswell, Annette Dunham, Ann McKillop, Timothy Kenealy, Nicolette Sheridan, Walter Wodchis

**Affiliations:** 1 Women’s College Hospital, Institute for Health System Solutions and Virtual Care, Toronto, Canada; 2 University of Toronto, Institute of Health Policy, Management and Evaluation, Toronto, Canada; 3 Bridgepoint Collaboratory for Research and Innovation, Lunenfeld-Tanenbaum, Research Institute, Sinai Health System, Toronto, Canada; 4 Health Policy and management, Canada research chair on health system design and adaptation, School of Public Health, Université de Montréal, CRCHUM, Montreal, Canada; 5 Université de Sherbrooke, Centre de recherche hôpital Charles-Le Moyne, Longueuil, Canada; 6 School of Population Health, University of Auckland, Auckland, New Zealand; 7 University of Auckland, Auckland, New Zealand; 8 School of Nursing, University of Auckland, Auckland, New Zealand; 9 Department of Medicine, University of Auckland, Auckland, New Zealand; 10 School of Nursing, Massey University, Auckland, New Zealand; 11 Implementation and Evaluation Science, Institute for Better Health, Trillium Health Partners, Toronto, Canada

**Keywords:** Realist evaluation, Realism, Complex health interventions, Mechanisms, Context, Implementation, Primary health care

## Abstract

**Background:**

The concept of “mechanism” is central to realist approaches to research, yet research teams struggle to operationalize and apply the concept in empirical research. Our large, interdisciplinary research team has also experienced challenges in making the concept useful in our study of the implementation of models of integrated community-based primary health care (ICBPHC) in three international jurisdictions (Ontario and Quebec in Canada, and in New Zealand).

**Methods:**

In this paper we summarize definitions of mechanism found in realist methodological literature, and report an empirical example of a realist analysis of the implementation ICBPHC.

**Results:**

We use our empirical example to illustrate two points. First, the distinction between contexts and mechanisms might ultimately be arbitrary, with more distally located mechanisms becoming contexts as research teams focus their analytic attention more proximally to the outcome of interest. Second, the relationships between mechanisms, human reasoning, and human agency need to be considered in greater detail to inform realist-informed analysis; understanding these relationships is fundamental to understanding the ways in which mechanisms operate through individuals and groups to effect the outcomes of complex health interventions.

**Conclusions:**

We conclude our paper with reflections on human agency and outline the implications of our analysis for realist research and realist evaluation.

## Background

The concept of “mechanism” has become a very popular analytic device in applied social science, and particularly in health services and policy research. This concept is closely associated with the rise of realist evaluation [1], which seeks to articulate what works, for whom, under what circumstances [2]. This general approach has been used in a variety of settings to contribute to better understanding how to implement research evidence and new models of health and social care, as illustrated by two recent reviews on the topic of mechanisms in the journal *Implementation Science* [3, 4]. However, the methodological guidance offered for researchers looking to apply the concepts and methodology of this kind of realism remains challenging to adapt to various topics and settings, particularly in relation to complex interventions in health care. We suggest that these challenges primarily arise from divergent interpretations and applications of the core concepts of realist approaches: mechanism and context.

The main progenitors of realist evaluation (and especially Ray Pawson) have come to define mechanisms in their more recent work specifically as the resources offered by program interventions and the ways in which those resources influence participants’ reasoning, thereby effecting program outcomes [2, 4, 5]. However, the broader literature defining and applying the concept of mechanism in various domains of applied social science has illustrated some limitations of this narrow definition of mechanism [1, 6]. Furthermore, even when keeping to the definition favoured by Pawson and Tilley, the concept has been taken up and applied in widely different ways in applied realist research; these differences are summarized in the background section of our paper [3, 4]. The apparent confusion about the concept of mechanism demonstrates the need for continued conceptual and methodological discussion on the topic. Clearly the concept has seemed to many investigators to promise a fruitful, stimulating direction of inquiry [3, 4, 7], yet the range of definitions and modes of usage still present in the literature may undermine the benefits of the concept.

Our large, interdisciplinary research team has experienced similar challenges in making the methodological concept of mechanisms useful in our study of the implementation of models of integrated community-based primary health care (ICBPHC) in three international jurisdictions (Ontario and Quebec in Canada, and in New Zealand). This paper reports on our experience in relation to both empirical research and the theoretical literature, advancing dialogue on the conceptualization and operationalization of mechanisms in realist research and evaluation. Specifically, we articulate various perspectives on the mechanism concept and illustrate its use in empirical research, examining what we consider to be two central points of contention in the realist community. The first pertains to the relationship between contexts and mechanisms, and the second relates to the nature of “reasoning” as a causal mechanism in realist-informed research.

In response to the first issue, we explore the possibility that the distinction between context and mechanism might be ultimately arbitrary, such that mechanisms occurring more distally in a causal chain that leads to a particular outcome become contexts for *subsequent* mechanisms occurring more proximally to the outcome of interest. In this way, the causes of outcomes of complex health interventions, policy programs, or any social action may be viewed as either contexts *or* mechanisms depending on where the analytic focus is located in a given causal chain. We acknowledge this is a complex chain of reasoning, and the ideas remain highly abstract. We provide an example from our study of the implementation of ICBPHC to make these ideas more concrete, and suggest that further thought is required about the assumed ontological difference between context and mechanism in empirical realist analyses.

For example, a policy is generally considered to constitute a feature of context for a particular intervention [2]; however, our analysis illustrates that a particular policy expressed an important mechanism early in the causal chain that eventually led to a particular positive outcome for patients (detailed later in this article). We believe it is inevitable that mechanisms appearing much earlier in a causal chain be grouped together and treated as context, and suggest that when and how this occurs depends on the theoretical assumptions and practical interests of the researchers carrying out the analysis. This should be done conscientiously and justified thoroughly in order to enhance the trustworthiness of the analysis [8, 9]. As opposed to being an argument *against* realist-informed research approaches, we suggest that understanding this point will empower realist researchers to move beyond lists of context-mechanism-outcome configurations and embrace a more theoretically and pragmatically satisfying explanatory data analysis.

In response to the second issue, we examine various ways of conceptualizing “reasoning” as a possible mechanism for achieving change, and examine its conceptual links to human agency in achieving particular program outcomes. Our research team began our methodological discussion by asking whether one could posit the existence of “organizational mechanisms” or “policy mechanisms” that influenced outcomes by means other than “resources and reasoning”, and that exist separately and apart from “context”. Our response to this question is that it depends upon how one conceptualizes “reasoning”. Is it a conscious, active process depending on intended human action? Or might “reasoning” also include the non-conscious (or at least less-conscious) rationality occurring beneath explicit awareness that causes an experienced driver to stop a car at a stop sign? If the latter example is also “reasoning”, then we agree that mechanisms may be considered to influence outcomes via their impact on human reasoning. Regardless of how readers choose to answer this question, we suggest that it is a point of contention in realist-informed research and demands further reflection and debate.

Ultimately we believe that realists have the responsibility to articulate mechanisms, contexts and outcomes with the utmost clarity in order to avoid the confusions that have characterized this methodological approach. As Margaret Archer (2000) explained:

“The direct implication then, is that social realists have to be a good deal more precise about [the] properties and powers of human beings, and how they emerge through our *relations* with the world, which cannot be narrowly construed as ‘society’, let alone as ‘language’, ‘discourse’ and ‘conversation’.” (p. 7, emphasis in original) [10]

This paper represents the effort of our interdisciplinary research team to be “a good deal more precise” about operationalizing the concept of mechanism in our research. We hope that it will be of some use to other research teams grappling with the challenge of articulating mechanisms and their relations to contexts in applied social science, and look forward to further discussion and debate on the matter.

### A brief tour of mechanisms

It is widely recognized in evaluation literature that the effort to identify “mechanisms” by which program interventions work predates Pawson and Tilley’s (1997) book, *Realistic Evaluation* [5]. Astbury and Leeuw (2010) suggest that the work of Chen and Rossi in 1987 and 1990 represent the first usage of the term mechanism as a means of “opening up the black box” of evaluation, explaining the reasons why programs cause particular outcomes [11–13]. However, the notion of using theory to build explanations in evaluation predates that; the work of Carol Weiss was already addressing theory as a strategy to explain why policy programs worked or not in the 1970’s [14]. But as Astbury and Leeuw (2010) suggest, it was not until Pawson and Tilley’s *Realistic Evaluation* that a systematic discussion of mechanisms was seen in the context of applied realist research and evaluation [5].

As might be expected in efforts to translate the work of a broad camp of theorists (philosophical “realists”) into methodological guidance, a variety of interpretations of central realist concepts have arisen over the past 20 years especially [15–17]. This includes the concept of mechanism, which has been at the centre of efforts to describe realist-informed methodologies. Here we provide a very brief overview of various definitions of mechanism that have appeared in realist applied social science, starting with the ongoing work of Pawson, Tilley and their realist evaluation colleagues.

### Mechanisms in Pawson and Tilley’s realist evaluation

Dalkin et al. (2015) [3] succinctly summarized the version of mechanism promoted by Pawson and Tilley in realist evaluation work as “a combination of resources offered by the social programme under study and stakeholders’ reasoning in response” (p. 3). This definition suggests that programs offer “resources”, which alter peoples’ “reasoning”, and that the relationships between these resources and the resultant reasoning of individuals constitute the mechanisms by which a program “works”. This restricts mechanisms to cognitive processes and the environmental stimuli that alter those processes. In *The Science of Evaluation: A Realist Manifesto*, Pawson (2013) explained this basic position as follows:

“Steadily and imperceptibly, successful programs recruit with care, create expectations, demand effort, offer sounding boards and encouragement, build and test resilience, accommodate setbacks, explain themselves, share execution and control, offer onward external continuation, and so on. All of these are mechanisms – they are resources capable of infiltrating the subjects’ reasoning... Their role is to support, enable, engender or catalyze the principal policy objective.” (p. 128)

In this way, mechanisms do not refer to actual activities that compose a program (such as “hosting inter-professional meetings” or “handing out transit tokens”), but to the description of *how those activities result in changes* to behavior, and as a result, in other program outcomes. This definition positions mechanisms specifically as descriptions of *how* specific program activities lead to specific changes in behavior, wherein individual behavior constitutes the relevant outcomes of policy programs. In their earlier work, *Realistic Evaluation*, Pawson and Tilley (1997) summarized this *process-oriented* view as follows: “A mechanism is not a variable but an account of the behavior and interrelationships of the processes that are responsible for the change. A mechanism is thus a theory” (p. 68) [5]. More specifically for realist evaluation, it is a theory of what causes changes in individual behavior.

Pawson spent an entire chapter outlining his realist theory of behavior change in his 2013 book, clarifying how mechanisms interact with the individual behavior change process. Pawson’s account of human behavior change is in large part based on Margaret Archer’s morphogenetic approach [10, 17, 18], wherein the reflexivity of individuals (conscious thoughts and reflections on their personal needs, wishes and the demands placed upon them) is taken to be central to understanding action and change. This is not to underestimate the influence of social structure, but simply to emphasize that it is reflexivity at the individual level that is most responsible for individual change. In the same way, according to Archer, reflexivity is also essential for understanding social reproduction and transformation. This perspective on the role of reflexivity (and “reasoning” specifically) in individual behavior change provides important context for the ways in which the mechanism concept has been used in Pawson’s version of realist informed research and realist evaluation. However, other commentators have envisioned different conceptualizations and applications of the mechanism concept; we summarize some of these perspectives next.

### Alternative views on mechanisms

One of the primary differences between the version of mechanism espoused by Pawson and Tilley and that described by other recent commentators relates to the appropriate level(s) of analysis; what changes (other than reasoning) as a result of the action of mechanisms, and how might these mechanisms be considered to “activate” their effects? A number of other realist researchers describe the possibility of mechanisms existing at levels of analysis beyond the individual’s reasoning [1, 3, 6, 11]. For example, in a political science context, Falleti and Lynch (2009) explain that

…mechanisms may occur at a variety of levels of analysis and in different types of contexts. And micro-level mechanisms are no more fundamental than macro-level ones. Some are individually based (adaptive expectations, rational choice), whereas others apply to collective actors (policy ratchet effects, layering, conversion), social systems (increasing returns, functional consequences), or both (policy drift). (p. 1150) [6].

Illustrating the possibility of identifying mechanisms at yet different levels of analysis, DeSouza (2013) explains that “it is possible to suggest that mechanisms in an action context are located within institutional structure, culture, agency and the relational properties between them” (p. 146) [19].

These definitions imply different understandings of the social constructs that may be implicated in explanations of mechanisms. Essentially, they suggest that a researcher’s definition of mechanism will depend on his or her underlying beliefs and interests in the analytically relevant units of analysis and components of the empirical case that relate to the primary object of a given study.

Further illustrating the challenge of the mechanism concept, Astbury and Leeuw (2010) suggest that “mechanisms are underlying entities, processes, or structures which operate in particular contexts to generate outcomes of interest” (p. 368) [11]. Here Astbury and Leeuw (2010) argue that mechanisms may be understood as (a) discrete objects (“entities”), (b) actions or movements over time (“processes”), and (c) relatively stable arrangements of social and/or physical environments (“structure”). Here the confusion seems to be about whether mechanisms are actual “things” (entities), or simply explanations about *how particular outcomes have been achieved* in the world.

Authors looking to explicitly incorporate and build upon the version of mechanism used by Pawson and Tilley have also demonstrated different interpretations of the concept. For example, in their description of Pawson and Tilley’s approach, Marchal et al. (2012) explained that

“In [Pawson and Tilley’s] view, both actors and programmes are rooted in a stratified social reality, which results from an interplay between individuals and institutions, each with their own interest and objectives. If all human action is embedded within such a wider range of social processes, then causal mechanisms reside in social relations and context as much as in individuals” (p. 195) [1].

This quotation suggests that mechanisms causing program outcomes may be considered to emerge from the ways in which various elements of a social system interact (i.e., as a process), and problematizes the clear-cut difference between “mechanism” and “context”. However, these authors also suggest that mechanisms “reside” in a particular place, perhaps creating confusion by oscillating between the view of mechanisms as processes and mechanisms as entities to be uncovered.

Dalkin et al. (2015) trace different understandings of mechanisms back to the philosophical roots from which they arose, acknowledging that Pawson and Tilley (1997) adopted a different understanding of mechanism than that found in other realist theory. These authors suggested that “mechanisms can have different meanings depending on the scope of the intended explanation” (p. 2) [3]. Providing an example, they explain that,

“structural mechanisms come to the fore if the social scientist is attempting to explain large-scale social transformations. If, however, the researcher is attempting to discover whether a particular fitness programme creates healthier participants, it can be assumed that key outcomes will result from the reasoning and responses of the participants” (Dalkin et al., 2015, p. 2).

This quotation by Dalkin et al. (2015) is instructive. It suggests that the specific uses of the concept of mechanism to build explanations of why particular changes are seen *will vary depending on the kind of change that is the primary focus* of a realist-informed study. If the changes are organizational, then organizational mechanisms will be sought. If they are individual, then individual mechanisms will be sought. Said differently, the kind of mechanisms that are the focus of a given analysis will depend upon the theoretical reference points of the research team carrying out the study.

### Context

The summary of how mechanisms have been conceptualized in literature on realist research and evaluation suggests that a brief commentary on context is also necessary in order to more completely clarify the relationships between the two concepts. Pawson (2013) does well to emphasize that context is a very challenging concept to pin down, that it has layers of relevance, and that it has real influence on both the nature and implementation of a social program. From our reading of the realist evaluation literature, we view context as being described in two distinct ways with relevance to our discussion here. First, context is described as the circumstances into which a program is implemented, being used as an explanation for why two or more programs that are ostensibly the same at the planning phase always end up appearing different after implementation. Second, moving beyond a simple description of circumstances, context is conceptualized as something that can be broken into meaningful segments and further specified to articulate the exact types or manifestations of context that serve to promote or inhibit the activation of mechanisms.

Pawson (2013) categorized contextual influences into four groups, including individuals (their characteristics and capacities), interpersonal relations (stakeholder relationships), institutional settings (rules, norms and customs), and infrastructure (social, economic, and cultural setting) (p. 37). Recognizing that it is very difficult to discuss a concept as diverse as context without simplifying into categories such as these, this method of grouping contextual influences does little to assist research teams in determining which contexts actually matter. Alternative approaches to conceptualizing context have focused on identifying the more proximal influences around a particular action or outcome of interest, and linking those influences through the development of chains that connect one contextual influence to another [20–22]. This approach moves away from the effort to connect a single context to a single mechanism, and instead examines the *links between* manifestations of context that matter for the focal phenomena in a research study, whether they be contexts located in a patient’s living room or contexts located across an entire regional health care system. In this way, researchers are able to develop a network of contextual influences that interact in their relevance on a given mechanism, as opposed to linking individual contextual influences to individual mechanisms.

This approach to identifying which contexts matter begins by looking at what outcomes are significant in the data, and tracing the mechanisms outward to understand what might have caused those outcomes to be achieved. This will resonate strongly with realist evaluators. However, we suggest the challenge arises in the effort to identify the range of significant causal links that lead to the outcome of interest, and deciding which links should be considered mechanisms and which should be considered contexts. We now turn to an empirical example, which will help to clarify this point.

## Methods

### Empirical case example: The iCOACH study

The insights presented in this paper were generated out of the experience of our research team working on a large cross-jurisdictional study of the implementation of ICBPHC. This study is referred to as the “implementing integrated care for older adults with complex health needs” (iCOACH) study, and involves case studies of three purposively selected cases in each of three jurisdictions: Ontario and Quebec (both in Canada), and New Zealand (for a total of nine cases). The overarching research question for this research program is, “What are the steps to implementing innovative models of ICBPHC that address health and social needs, and improve outcomes for older adults with complex care needs?” The ultimate objective is to develop a systematic guide to the design, implementation and scaling up of innovative models of ICBPHC that improve outcomes for patients and carers.

In our research program, we acknowledge that there are many links in the causal chain that eventually leads to better experiences and outcomes for patient and carers, potentially including new clinical collaborations, organizational structures, leadership styles, and policy frameworks. Our analysis focused on understanding the mechanisms that connect the most salient elements of that causal chain, using a consistent definition of mechanism as a starting point but acknowledging that it might need revisiting throughout the analysis. Our starting point was thus to define mechanism as “a *causal explanation* for why a particular action or intervention had an observed outcome, whether that outcome be a new organizational form, a new way of approaching a problem, or any other type of structure or experience”.

Two points are worth noting up front. The first relates to the nature of the cases selected (loosely defined as “innovative models of ICBPHC”). Identifying “exemplars” of ICBPHC proved immensely difficult, and the process has been documented in detail elsewhere (see Kuluski et al., 2017) [23]. One key lesson learned early in the selection process is that exemplar models of ICBPHC in the settings we studied do not generally exist as clearly defined programs with well-established boundaries. Instead, they are constantly evolving collaborative endeavours in which it can be very difficult both to determine a clear starting point and specify the relevant participants. These early challenges helped to shape our research approach and the insights presented here.

The second point is that our methodology represents realist-informed research, not realist evaluation per se. The methodology of realist evaluation outlines a set of theories and methods applicable for the evaluation of a range of well-defined programs [2]. As we discovered, models of ICBPHC across the three jurisdictions could not be described as “well-defined programs”, and instead included a variety of providers organized in a variety of ways, and responding to a variety of policy realities. As such, we took a more inductive, open-ended approach to our analysis that sought to document how and why the implementation of these models unfolded in particular ways, as opposed to intending to evaluate their effectiveness using formal methods of realist evaluation.

Building on these realities, our team conducted interviews with policy stakeholders in each jurisdiction, as well as organizational leaders, health care providers, patients and unpaid carers in each individual case (over 250 interviews overall). We also completed observations where possible, and analyzed important documents identified by participants including websites, annual reports, and strategic plans. Although our analysis is ongoing, the insights presented in this paper have substantially shaped the analytic approach of our research program. Here we present select data from a single case in Ontario, Canada, with the intention of illustrating our conceptualization and application of a realist approach to research.

### The case

**Fig. 1:**
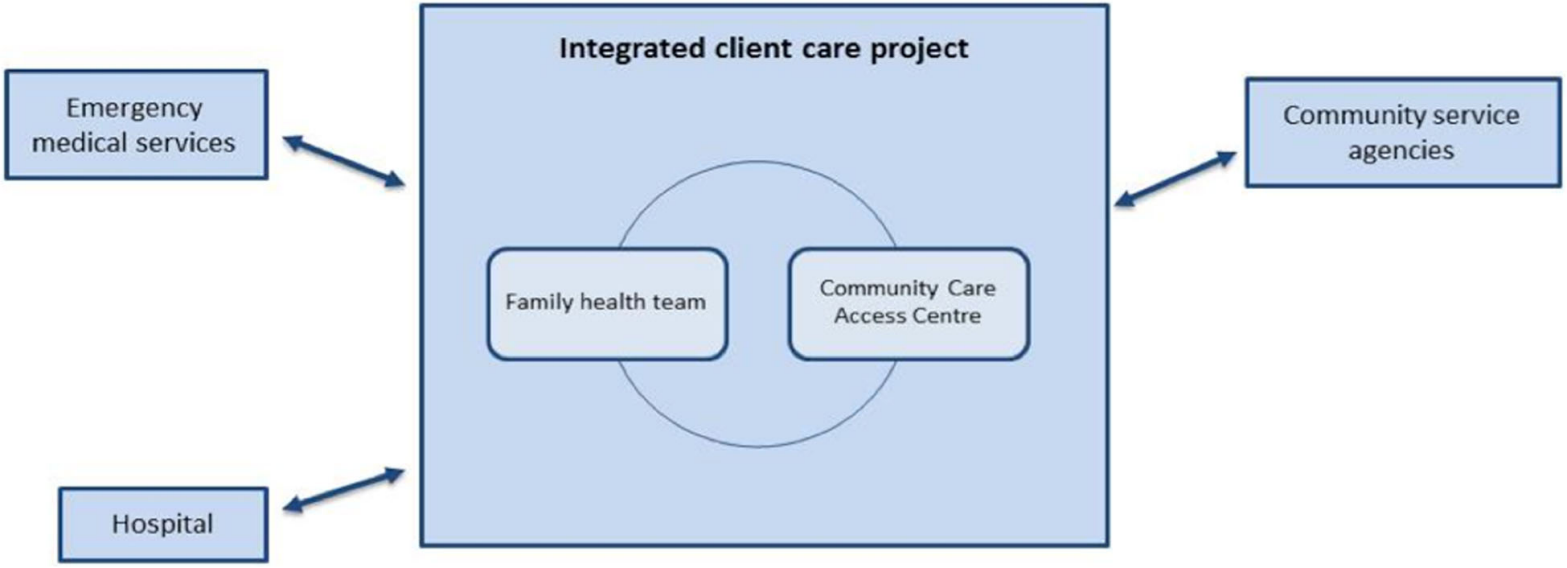
Schematic of Integrated Client Care Project Case (Ontario), adapted from Breton et al. (2017) [24]

The case from which we present data here was originally identified as a formal collaboration between two organizations: a home and community care commissioner (a community care access centre, or CCAC) and an interprofessional primary health care centre (a “family health team” or FHT). Their joint collaboration was referred to as the “integrated client care project” (ICCP). Through the research process we discovered the importance of a variety of other collaborating organizations, most notably the local hospital (located very near the family health team) and a community services agency. Although our intention was to study the formal program that brought the original two organizations together in a collaborative model of ICBPHC, we discovered that the effort to achieve integrated care in this case pre-dated their formal collaboration. A process of layering programs on top of one another and building relationships over time had led to a comprehensive approach to community-oriented primary care that emphasized both inter-professional and inter-organizational collaboration. A visual schematic of the organizations in this case is presented in Fig. 1 (reprinted with permission from Breton et al., 2017) [24].

The data we present below are not intended to provide a comprehensive report of the mechanisms and contexts that led to the outcome of this novel model of integrated CBPHC. Instead, we present data simply to illustrate our approach to applying the concept of mechanism, and to raise methodological questions that advance dialogue in realist research and evaluation.

## Results

### Tracing mechanisms through the case

In the effort to articulate a collection of mechanisms operating in our case example, we first begin with outcomes. Following this approach we articulate the outcomes achieved for patients and unpaid carers, describing the specific benefits experienced by these participants as a result of the model of ICBPHC we are studying. We then build explanations backward by describing the mechanisms that caused those outcomes to be achieved. This means understanding the actions of various people who make up the case, and the organizational, professional, and policy contexts that activate and motivate their work. Synthesizing these various elements of our case description culminates in the identification of mechanisms that brought the program outcomes into being. We then comment on relevant contexts.

### Outcomes for patients and carers

We have documented a variety of outcomes for patients and carers arising from the unique models of care included in our comparative case studies, which will be reported in detail elsewhere. Although our cases did not achieve these outcomes for every patient every time, the instances where positive outcomes were achieved provided the opportunity to articulate what worked, for which people, and under which circumstances. The outcome we address now is the experience among patients and carers that they have a health care team they can count on; that providers are responsive and work together to ensure patient needs are met. This outcome represents the demonstration by health care providers over time that they are reliable and will respond to patients as needed, leading to beliefs among patients and carers that they can count on the health care team. This outcome was identified through qualitative interviews with patients and carers.

One woman who is the primary carer for her husband (who is living with multiple chronic conditions) described this outcome as follows:

“Doctor (name), just about does everything and- (name)… the coordinator, they all come in and they’re the ones that do all the phoning and the people just call here to make appointments and-and they come in… And then there is, um, if I need a change of mattress, or anything like that, they come in, you know, and they make sure that his mattress is okay for him, or [if I] have to buy another one, uh, [they] buy it… So there’s always (sighs), they’re always there, and they all seem to know what’s going on with [patient name], and they come when they’re needed. If I need any prescriptions or anything, I phone into the doctor’s office, they fax it in, and it comes to me.” [SC 05]

This quotation demonstrates a number of actual instances in which the health care team responded quickly and comprehensively to the needs of this patient and carer. The carer feels comfortable knowing that the health care team is there to support her and her husband. In our assessment, this reliability and responsiveness of health care providers represents an important outcome for models of ICBPHC.

### Mechanisms at the health care provider level

What were health care providers doing that enabled them to be so responsive, and present such a unified team-based service to this patient and carer? The health care providers described by this carer were a part of a “home visit team” at our case site, created under the remit of the Integrated Client Care Project described earlier. The home visit team is an interprofessional group of health care providers who provide ongoing management of patients with multiple complex conditions, and have the authority to make home visits if deemed necessary by the group. They have regular meetings to discuss their shared patients, include providers from across three organizations, and have a mandate to be flexible and creative in their response to patient needs.

Two closely linked mechanisms at the health care provider level are most directly associated with the outcome just described. The first mechanism is the *commitment to a shared set of operating principles* among the health care providers regarding the provision of comprehensive care in peoples’ homes. The second mechanism is *the development of interpersonal relationships among the health care team* that enabled them to share information, coordinate interventions, and provide more comprehensive services to patients than would otherwise be possible.

The shared operating principles among the home visit team were established by the providers themselves early in the development of the program, as they planned the best ways to meet the needs of patients in their community. One health care provider described the role of the “home visit team” as a result of those earlier conversations as follows:

“So, the home visit team …. [now] we do rounds once a week to talk about patients who may have questions or concerns or troubleshoot with. And then as part of the home visit team, we all have our own clients or patients that we see, that we’ll see them regularly and more often as needed just to provide primary care to them in their home. And then if something urgent comes up, we try to address those.” (SE-03)

This vision for the purpose and function of the home visit team was shared among its members. However, the health care providers who make up the home visit team are not all employed by the same organization. The collaboration between the FHT and the CCAC was particularly important, as described by one FHT employee:

“And certainly our home visit program, we partner very closely with CCAC - we have 2 coordinators that work onsite with us that are usually assigned to our patient roster. So, they are fantastic resources and they help us to sort out, if they need any PT/OT, any kind of things within the home. So, that’s really helped us to kind of round out the resources and services for our patients within the home, without them I don’t know what we would do.” (SE-10)

Engaging in the effective coordination of care for patients with such complex needs was recognized by team members as requiring frequent communication for the exchange of information and expertise among health care providers. The establishment of interpersonal relationships among the team members meant that such communication could happen much more easily than if they did not already know, encounter, and respect one another. One participant described this as follows:

“Like, and that’s a part of why we brought the CCAC people in-house because that geographic closeness just makes a difference, right? Like, when you’re around people, having lunch with those people or whatever, it’s that human social contact that allows collaboration to be so much more easy and natural.” (SE-06)

The shared operating principle of meeting patients’ needs in their homes, and the establishment of relationships that enabled team members to meet those needs, are mechanisms at the health care provider level that helped to achieve the patient and carer outcome described in this example. It is worth repeating that these are only two mechanisms building the causal explanation for the outcomes observed, and others certainly exist. Here we have not mentioned the obvious clinical interventions and interpersonal interactions between health care providers, patients, and carers without which no outcomes could be achieved at all. The mechanisms reported here are important findings, but only represent the first step in our analysis; the next challenge is to articulate the features of the organization and its leadership that activated these mechanisms in our case.

### Mechanisms at the organizational level

The leadership of the FHT, the hospital, and the CCAC had agreed to establish a particular structure and approach to the management of the home visit team. Specifically, the leaders established the expectation that CCAC care coordinators would have office space at the FHT, and would participate in meetings and care planning of FHT patients. The hospital leadership committed resources to ensure information sharing and collaboration between hospital staff and the FHT home visit team. But what were the mechanisms that enabled the health care providers to establish such firmly held operating principles, and to build the relationships that drove the more personalized and responsive delivery of care to patients?

Two mechanisms at the managerial level are particularly relevant in this instance: *enabling self-determination among the health care provider team* and *encouraging relationship-building through co-location of health care providers*. Elaborating on the first mechanism (enabling self-determination, in the sense that health care providers had control over determining which services they would provide), one organizational leader explained,

“there was a lot of liberty given to the frontline staff. So it wasn’t a top, top-down approach at all from a CCAC perspective, which really, um, was very refreshing. And it allowed [them] as a team to really build this model together… It was really driven by the frontline workers, um, more or less, you know, with a little bit of guidance from my end.” (SE-13)

Here this leader articulates a managerial strategy in which health care providers were enabled to build the details of the home visit program based on what they observed were the needs of their patients. However, this approach would not have been successful if it was driven by only one leader; there were three organizations involved in the home visit team, and all three needed to agree with this direction. The hospital leader explained,

“[The program structure] is a bit loose, and we’ve kept it that way. And one of the reasons, as a hospital, as a leader, I’m not really that keen on imposing on that group [is] because they need to have the freedom to adjust to what the community needs. And the discussions are more about the dialogue… If you were to ask me what the vision is of that group, it is to adjust and adapt to the changing needs of the patients and the providers that are caring for them, not necessarily ‘this is what they need to do’. Because by definition, these patients aren’t typical. We’ve already determined that. And our goal is to come up with [a] process that deals with the outliers, not the standard processes.” (SE-04)

The managerial and leadership approach articulated by these leaders was based upon two central mechanisms. By mandating co-location of CCAC and FHT health care providers, and then enabling the self-determination of the design of the home visit program by providers, leaders fostered a strong commitment to a shared set of operating principles and the establishment of interpersonal relationships among health care providers. But the explanation does not end here. What enabled these organizational leaders to adopt this particular collaborative approach to managing this new initiative?

### Mechanisms at the policy level

The organizational leaders at the hospital and the FHT had pre-existing relationships, which had been in place for approximately 5 years. This was in part the result of the close proximity of the hospital and the FHT; they were located directly across the road from one another. However, in driving this integrated model forward in the local area, the FHT leaders acknowledged that it would be essential to interface more closely with home care services too. Although the CCAC had a strong reputation for collaboration, the leadership of the FHT and the CCAC had not yet worked together on projects requiring such shared commitment and resources.

During the time that the FHT was pioneering this more integrated model in the local area, new legislation was passed in Ontario called the Excellent Care for All Act (2010). This new policy set directions for health care organizations, beginning with hospitals, intending to enhance the focus on quality improvement and patient engagement. Although the policy did not include specific stipulations for FHTs, the introduction of the policy was interpreted as signifying a trend in expectations from the Ontario Ministry of Health and Long Term Care that all health care organizations must work more collaboratively and transparently toward achieving higher quality patient-centred care. A leader at the FHT described the impact of the Excellent Care for All Act (2010) as follows:

“Home care was our third major party to come along, and I think there - it was just, um - that was actually really… the good luck of the Excellent Care for All Act really coming right at the time when we were thinking about improving care for patients and making patients the centre of care. So that was exactly what the Virtual Ward was [a precursor of the Home Visit program], um, intending to do. And so a major healthcare policy shift allowed us to really have the driver… you know, at hand to also bring CCAC onboard, because it aligned, um, with, major healthcare policies.” (SE-13)

The mechanism embedded in the announcement of the Excellent Care for All Act (not in the actual legislation, but instead in its interpretation by leaders) was *providing justification for inter-organizational collaboration*. Health care organizations face many competing priorities, and organizational leaders must sort through the challenges and opportunities faced by the organization to decide strategic priorities and directions. The announcement of this new policy signalled to the organizational leaders that an enhanced focus on quality and collaboration would become increasingly important in the years ahead, providing justification for the FHT to ask a neighbouring health care organization (the CCAC) to expend additional resources on a new collaboration.

### Contexts versus mechanisms

Does the Excellent Care for All Act (2010) represent a *context* for the ultimate outcome of patients and carers feeling they have a health care team they can rely on, or a causal *mechanism* underlying the development of an inter-organizational collaboration among the involved organizations? Or both? We contend that this policy acts as both a mechanism and a context depending on the specific perspective from which the case is being analyzed. If the focus is on the most proximal mechanisms leading to improved outcomes for patients and carers, then the policy represents a context. However, if the casual pathway is being clearly laid out in an attempt to understand in detail how this policy relates to the outcomes observed, then the announcement of the policy itself represents one mechanism in a chain of causal relationships leading to the outcomes of interest.

**Fig. 2:**
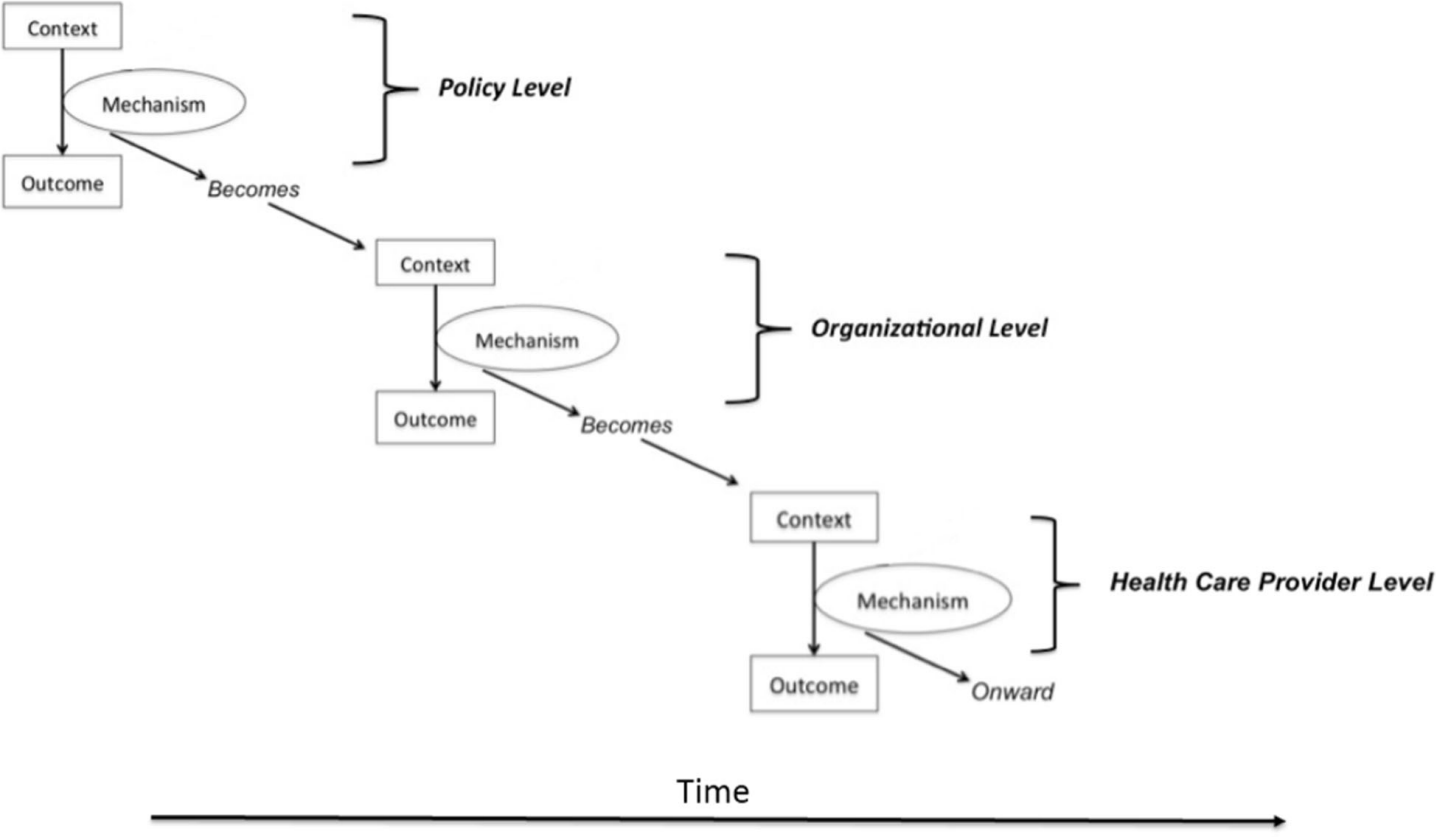
Depiction of relationships between contexts and mechanisms. Mechanisms may become contexts at more proximal locations along a causal chain. This figure is inherently limited by being presented as over-linear; we acknowledge that the action of mechanisms and contexts take place in less linearly defined ways

The same could be said for all of the mechanisms outlined in our case report. When viewed as mechanisms (as presented here), each comes along with a range of contextual influences that enabled the mechanisms to have their causal effects. For example, the mechanism of “providing justification for inter-organizational collaboration” may not have been effective in the absence of committed leadership, pre-existing relationships between the FHT and the hospital, and the geographic proximity of the FHT and the hospital. In this instance, this range of contexts were the ones that “mattered”. The mechanism of “enabling self-determination among the health care provider team” may not have been effective in the absence of well-trained health care providers who understand the needs of their patients. But these features of time, geography, relationships, and knowledge could just as well be described as having causal influence on activities observed in our case, thereby qualifying as causal mechanisms over and above features of context. From our experience of this analysis, making these decisions depended on a collaborative negotiation between theoretical perspectives and pragmatic demands of the issue at hand. Figure 2 provides a visual depiction of the relationship between contexts and mechanisms as we describe it here.

The point we are trying to make with our partial report of the analysis of this case is that it is up to us as a research team which features of our case we describe as “mechanisms” and which features we describe as “contexts”. A primary goal of this paper is to raise the attention of those engaging in realist-informed research to the analytic challenge of differentiating mechanisms and contexts. We suggest many features of our case could be described as both mechanisms and contexts (and likely *act as both* for different causal chains). Sorting out why and when to present various features of our case as one or the other is an analytic decision to be tracked and accounted for by the research team.

## Discussion

### Mechanisms, reasoning and human agency

Given this empirical example, we now turn to addressing the ways in which mechanisms affect human action in more detail. This section responds to the second of the two issues identified in our introduction as central points of contention in realist-informed research: the relationship between reasoning, human agency, and causal mechanisms.

Research and theory on the causes of human action have dominated a number of disciplines over the past century [25], including psychology [26, 27], sociology [28, 29], anthropology [30], economics [31], philosophy [32], and others. Applied research in health care relies in many ways on the insights of these disciplines, incorporating assumptions about how humans act in the world and what brings about changes in human behaviour [33]. Closely linked to this question is the ongoing debate about what constitutes the human subject, and these lines of theoretical dialogue come together in the conceptualization of *human agency* [34]. The nature of human agency, and its relationship to “context”, is at the root of realist ideas about the mechanisms by which programs lead to outcomes [2, 5, 35].

Our analysis and ongoing discussion among members of our research team eventually arrived at the topic of agency. Specifically, we came to agree that intervention mechanisms bring about changes in outcomes by acting in and through human agents. More challenging to bring to the surface of our discussions was the issue of the extent to which conscious, self-aware reasoning drives human agency in comparison to non-conscious, habitual forms of thought and action. Summarizing this debate is instructive in the effort to understand the ways in which mechanisms relate to outcomes in realist-informed research.

Pawson (2013) outlines a model of the ways in which programs lead to behavioural changes among participants, stating that “at all stages program participants are choice-makers” (p. 128) [2]. The model proposes that people move through various “state[s] of thinking” as they engage in behavioural change (p. 128), beginning with “disaffection”, then on to “self-doubt”, and then finally on to “adoption” and “conversion”. But in what ways are these to be considered “states of thinking”? In the mechanism of “committing to a shared set of operating principles” among health care providers, we are not suggesting that members of the health care team followed a simple, straightforward, self-aware process of behavior change. Instead, non-conscious elements related to habits of thought and professional practice, tacit expectations of other team members, implicit assumptions about professional obligations, and other features of everyday human activity that lie beneath conscious awareness would have played fundamental roles. In this way, the development of this new way of working through a shared set of operating principles would have been developed as much through unconscious expectations as through conscious efforts to change.

In our case health care providers actively engaged in sharing information with other providers, asking probing questions, seeking advice, engaging in professional debate, and over time developed a shared set of expectations related to how they would provide care to their patients. Some of this process would certainly have included conscious, self-aware, rational thought, and other elements of the process were more habitual and non-conscious, pertaining to the embodied development of something akin to a team culture. In their interdisciplinary review of the concept of human agency, Emirbayer and Mische (1999) explained:

“While routine, purpose, and judgment all constitute important dimensions of agency, none by itself captures its full complexity. Moreover, when one or another is conflated with agency itself, we lose a sense of the dynamic interplay among these dimensions and of how this interplay varies within different structural contexts of action.” (Emirbayer and Mische, 1998, p. 963) [34]

Human agency goes far beyond the rational thought and self-aware decision-making that some imagine as being the central driver of behavioural change [10, 29, 36]. Building on the work completed by Pawson and Tilley in articulating their approach to behavioural change, we suggest that a more sophisticated understanding of how mechanisms influence reasoning (both conscious and non-conscious) will strengthen the insights offered by realist-informed research, and will clarify the relevance of the mechanism concept.

Some realist researchers have of course already acknowledged this important insight, and have built a clear recognition of non-conscious forms of reasoning into their analysis. For example, Jagosh et al. (2015) outline the centrality of trust to the function and outcomes of community-based participatory research, describing trust as a phenomenon that is built and maintained through a complex collection of conscious (explicit partnership building) and non-conscious (by association with others or reputation) mechanisms [37]. Their analysis acknowledges the interplay between routine, purpose and judgment that is emphasized by Emirbayer and Mische (1998) above.

Making room in realist analyses for the full scope of ways in which human agency is enacted in the world is particularly important for understanding both *how mechanisms lead to change* and *why context matters*. Mechanisms can be effective in changing behaviour without appealing to rational decision-making. Context can shape human action by generating changes to team culture. This broader scope of human agency enables realist analyses to be “a good deal more precise” in describing the work of mechanisms and contexts, enabling more sophisticated and clearly stated analyses. It is our hope that the realist community can incorporate these insights into future research and evaluation, boosting the clarity with which we understand the ways in which complex health interventions have their impact on improved outcomes for patient, carers, and health care systems.

## Conclusion

In this paper we have provided a summary of the theoretical literature defining mechanisms in realist analysis, and have proposed two key points of theoretical contention that pose continued challenges for realist-informed research. First, we raised the possibility that mechanisms may also act as contexts in any individual intervention, and that researchers must judiciously and clearly articulate why certain influences are treated as contexts and others as mechanisms in a given analysis. Second, we highlighted the existence of various perspectives on the drivers of human agency, and advocated for greater clarity in articulating how mechanisms influence human action through a more nuanced view of agency. We encourage further discussion and debate on these issues, and hope that other researchers applying a realist approach to research and evaluation will find practical value from the discussion presented here.

## Abbreviations

CCAC: Community Care Access Centre
FHT: Family health team
ICBPHC: Integrated community based primary health care
ICCP: Integrated client care project
iCOACH: The integrating care for older adults with complex health needs study
